# Ultra-wideband microwave absorber by connecting multiple absorption bands of two different-sized hyperbolic metamaterial waveguide arrays

**DOI:** 10.1038/srep15367

**Published:** 2015-10-19

**Authors:** Xiang Yin, Chang Long, Junhao Li, Hua Zhu, Lin Chen, Jianguo Guan, Xun Li

**Affiliations:** 1 Wuhan National Laboratory for Optoelectronics, Huazhong University of Science and Technology, Wuhan 430074, China; 2 State Key Laboratory of Advanced Technology for Materials Synthesis and Processing, Wuhan University of Technology, Wuhan 430074, China; 3 Department of Electrical and Computer Engineering, McMaster University, 1280 Main Street West, Hamilton, Ontario, L8S 4L8 Canada

## Abstract

Microwave absorbers have important applications in various areas including stealth, camouflage, and antenna. Here, we have designed an ultra-broadband light absorber by integrating two different-sized tapered hyperbolic metamaterial (HMM) waveguides, each of which has wide but different absorption bands due to broadband slow-light response, into a unit cell. Both the numerical and experimental results demonstrate that in such a design strategy, the low absorption bands between high absorption bands with a single-sized tapered HMM waveguide array can be effectively eliminated, resulting in a largely expanded absorption bandwidth ranging from 2.3 to 40 GHz. The presented ultra-broadband light absorber is also insensitive to polarization and robust against incident angle. Our results offer a further step in developing practical artificial electromagnetic absorbers, which will impact a broad range of applications at microwave frequencies.

Microwave absorbing materials and microwave absorbers have important applications in various areas such as stealth, camouflage, and antenna^1^,^2^,^3^. In the past few years, great efforts have been devoted to the construction of highly efficient, wideband absorbers in the radio spectrum, especially in the low frequency regime of 2–18 GHz^4^. Among all the microwave absorbing materials, those based on ferrites and magnetic metals exhibit the largest absorption bandwidth, and have been demonstrated to be capable of absorbing more than 85% of the incident electromagnetic (EM) waves over the 8–18 GHz range^5^,^6^. However, it remains a great challenge to enhance the 2–8 GHz absorption because of the magnetic resonance nature and Snoek’s limit^7^,^8^. Metamaterial perfect absorbers (MPAs)^9^,^10^, typically comprised of dielectric thin-films sandwiched by a metallic split ring resonator and cutting wire, can show near 100% absorbance at the resonant frequency, but they generally exhibit a narrow absorption bandwidth^9^,^10^. To extend the working bandwidth of the MPAs, considerable effort has been dedicated to simply blending various resonators together. In such a way, the absorption bandwidth cannot be widened effectively because of the limited number of resonators that can be used in practical situations^11^,^12^,^13^,^14^,^15^,^16^,^17^,^18^. Recently, Guan’s group has constructed a lightweight, broadband two-layered hybrid absorber with 90% absorption in the whole frequency range of 2–18 GHz by integrating non-planar metamaterials with traditional magnetic absorbing materials^19^. To reduce the large effective thickness and relatively complex structure of such two-layered hybrid absorbers, they subsequently have designed and experimentally realized an ultra-broadband patterned microwave absorber with more than 90% absorption in the frequency range of 4–40 GHz by taking the advantages of multiple λ/4 resonances and the edge diffraction effect^20^.

Hyperbolic metamaterials (HMMs), typically consisting of a metal/dielectric multilayer, are promising for a variety of applications including indefinite cavities^21^, spontaneous emission enhancement^22^, and optical hyperlenses^23^,^24^,^25^,^26^. By using the hyperbolic dispersion curve, the authors have previously proposed and demonstrated far-field optical super-resolution imaging based on HMM medium^25^,^26^. Quite recently, HMM waveguide array has also been further employed to achieve broadband strong absorption of incident EM wave due to the large attenuation of slow-light mode^27^,^28^,^29^,^30^,^31^. However, to the best of our knowledge, merely slow-light of fundamental waveguide mode has been utilized, and hence the resultant absorption bandwidth is highly restricted. In this article, we have constructed an ultra-broadband microwave absorber by integrating two different-sized tapered HMM waveguides, each of which works at wide but different multiple absorbing bands, into a unit cell. The total absorption bandwidth can be effectively widened by connecting different absorption bands of different HMM waveguides by properly selecting the geometrical parameters for each waveguide. We demonstrate, numerically and experimentally, that such an absorber has the capability of working within an ultra-wide absorption bandwidth (2.3–40 GHz) with high absorption, good polarization independence and tolerance to incident angle.

## Results

### A single-sized tapered HMM absorber

**Figure 1:**
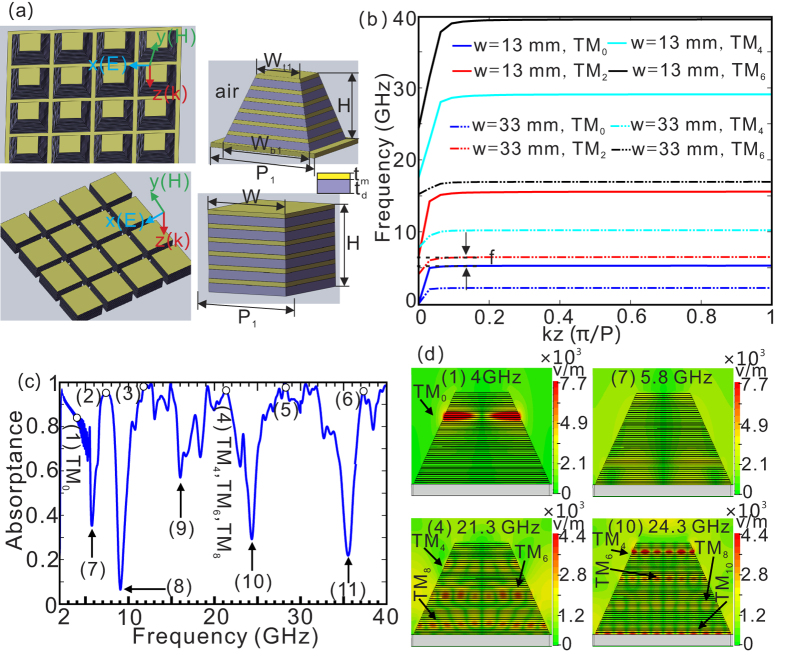
(**a**) Schematic of single-sized tapered HMM and uniform HMM waveguide arrays. Each HMM waveguide consists of alternating metal and dielectric layers with thickness denoted as 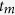 and 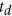, respectively. The top and bottom widths of tapered HMM waveguides are marked as 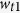 and 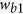. The lattice constants along x and y-direction are equal 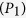. (**b**) The dispersion relations of uniform HMM waveguide arrays with two widths of 13 and 33 mm, respectively. The lattice constants along x and y-direction are all set to be 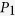 = 34 mm. *P* represents the lattice constant 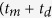, 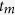 = 0.04 mm, 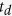 = 0.2 mm) along z-direction. (**c**) The simulated absorption curves of the single-sized tapered HMM waveguide array with height of *H* = 11.52 mm (48 pairs of alternating copper/FR4 layers) on a copper substrate. The insets (1)–(6) and (7)–(11) represent high, and low absorption bands, respectively. (**d**) The simulated amplitude distributions of electric field 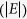 at (1), (4), (7), and (10) in (**c**).

Figure 1(a) shows the schematic of single-sized tapered HMM and uniform HMM waveguide arrays. For the tapered HMM waveguide array, the absorption frequency range is determined by the top width, bottom width, and the thickness filling ratio of the metal layer. By choosing a large difference between the top and bottom widths, the single-sized tapered HMM absorber can in principle operate over a wide frequency range throughout the visible, near-infrared, mid-infrared, terahertz, and microwave spectral regions. With the same top and bottom widths of the HMM waveguide array, the absorption spectrum of the HMM absorber would undergo a red-shift for a much bigger thickness filling ratio of metal layer. Here, the top and bottom widths of tapered HMM waveguides are set to be 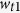 = 13 mm, and 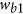 = 33 mm, respectively. The lattice constants along x and y-direction of the single-sized tapered HMM waveguide array are all set to be 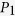 = 34 mm. And the lattice constant along z-direction is assumed to be *P* = 0.24 mm 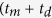, 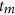 = 0.04 mm, 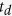 = 0.2 mm). Assuming the width grade of the tapered HMM waveguide array [left upper side of Fig. 1(a)] is sufficiently small, it can be deemed as a series of uniform HMM waveguide array [left down side of Fig. 1(a)]. The dispersion relations of uniform HMM waveguide array for different widths (*w* = 13, and 33 mm) are presented in Fig. 1(b), which clearly shows there exists multiple even waveguide modes for different widths. In Fig. 1(b), the horizontal axis represents the propagation constant, which is normalized to the lattice constant along z-direction. Recent study has demonstrated that the HMM waveguide array can simultaneously support the forward mode and backward mode with proper design of the structural parameters^29^,^30^. As the light frequency approaches the cutoff frequency, the two modes degenerate at a certain width of HMM waveguide, leading to a significant reduction of light velocity. Due to the large attenuation of the slow-light mode, HMM waveguide array has been used to construct broadband absorbers. The underlying physics behind the absorption efficiency is closely related to the photonic density of states^32^. A much higher photonic density of states will result in bigger absorption efficiency. Slow light occurs if light frequency approaches the cutoff frequency, and hence significantly enhance the photonic density of states, which leads to a strong absorption of EM wave. By utilizing the tapered HMM waveguide array with the top and bottom width as 13 and 33 mm, a high absorbance ranging from 2.3 to 5.2 GHz can be reached [(1) in Fig. 1(c)], corresponding to the frequency range of TM_0_ slow-light mode [Fig. 1(b)]. The TM_0_ mode propagates along the tapered waveguide, and finally localizes at a specific position due to significant reduction of group velocity [see (1) in Fig. 1(d) as an example]. It also presents more high absorption bands above 5.2 GHz [see (2)–(6) in Fig. 1(c)], which can be attributed to the excitation of slow light of higher order TM modes. As can be seen from (4) in Fig. 1(d), the electric field at 21.3 GHz is mainly localized at three different positions, associated with the cutoff frequencies of TM_4_, TM_6_, and TM_8_ modes, predicted by the dispersive relationship.

Unfortunately, there are also some low absorption bands between the high absorption bands [(7)–(11) in Fig. 1(c)]. The reason can be explained as follows. Firstly, there exists a frequency range [denoted by Δ*f* in Fig. 1(b)], where the tapered HMM waveguide array does not support any slow-light mode. Consequently, most of EM wave that is coupled to the structure will be reflected back into the air [see (7) in Fig. 1(d), where the electric field can not be localized at any place in the structure], leading to a low absorption band (7). Secondly, some slow-light modes occur by chance at the bottom of the HMM waveguide array, hence a portion of EM wave will be reflected back into the air by the metal substrate, forming several low absorption bands (8)–(11). It can be given a further explanation that the slow-light modes cannot confine the light well comparing with the conventional single-layered absorbers^15^,^16^,^17^. As the slow-light modes occur near the bottom of the HMM waveguide array, light can still propagate and then be reflected by the metal substrate. The weak confinement of light will finally cause the low absorptance, which is different from those conventional single-layered absorbers^15^,^16^,^17^. Though the resonance of the conventional single-layered absorbers also happens at the metallic bottom layer, the strong confinement of electric and magnetic field between the metallic bottom and pattern can absorb most of the EM waves instead of reflecting them. The low absorptance at 24.3 GHz [(10) in Fig. 1(c)] can be inferred by the electric field distribution, where the TM_10_ mode is located near the bottom of the structure [see (10) in Fig. 1(d)]. We have noted in previous study on tapered HMM absorber design, only the first high absorption band (1) caused by TM_0_ slow-light mode was used, inducing a relatively narrow absorption bandwidth^27^,^28^,^29^,^30^,^31^.

### Ultra-broadband absorber based on two different-sized tapered HMMs

**Figure 2:**
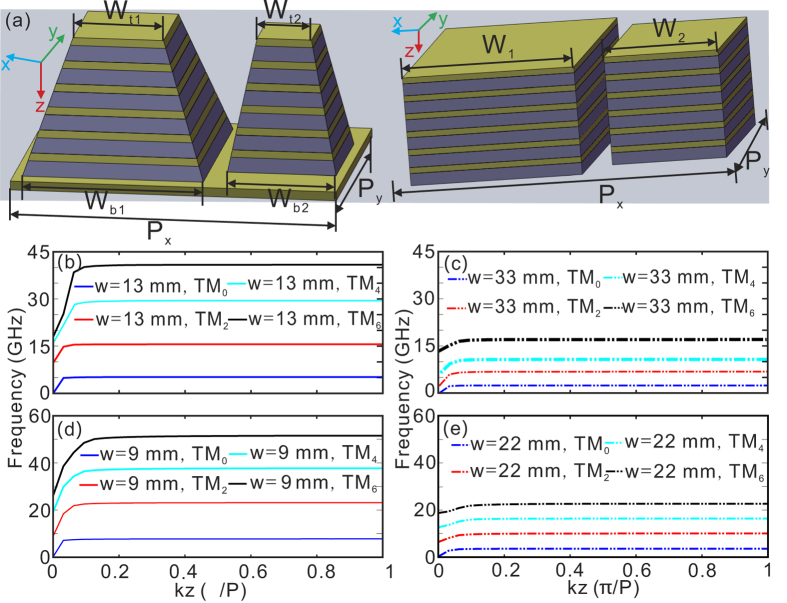
(**a**) The schematic of unit cells of tapered and uniform OS-HMM waveguide arrays. (**b**,**c**) The dispersion relations of the uniform O-HMM waveguide for two widths of (**b**) 13, and (**c**) 33 mm, while the widths of S-HMM waveguide are assumed to be 9 and 22 mm. (**d**,**e**) The dispersion relations of the uniform S-HMM waveguide for two widths of (**d**) 9, and (**e**) 22 mm, while the widths of O-HMM waveguide are assumed to be 13 and 33 mm. The lattice constants along x-direction and y-direction are assumed to be 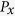 = 57 mm, and 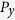 = 34 mm, respectively. All the other parameters are the same as those in Fig. 1.

In order to expand the absorption bandwidth in the HMM absorber, the low absorption bands (7)–(11) in Fig. 1(c) should be eliminated. We further consider adding another smaller tapered HMM (S-HMM) waveguide which is expected to exhibit high absorptance in these low absorption frequency bands (7)–(11) in Fig. 1(c), to construct a new absorber unit [tapered OS-HMM waveguide array, left side of Fig. 2(a)]. The top and bottom widths of the tapered S-HMM are set to be 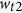 = 9 mm, and 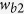 = 22 mm, respectively. The alternating tapered O-HMM and S-HMM waveguides are arranged with the lattice constant 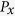 = 57 mm along x-direction, whereas the period of O-HMM waveguides along y-direction is 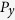 = 34 mm (also applys to the period of S-HMM waveguides in this direction). For a two different-sized uniform HMM waveguide array [right side of Fig. 2(a)], the dispersion curve of the original HMM (O-HMM) waveguide can be influenced due to the interaction of waveguide modes. We have noted the previous work on comparison of dispersion curves for a single unit HMM waveguide and a periodic HMM waveguide array, indicating that the dispersion curve can be slightly altered owing to the weak coupling between adjacent HMM waveguides^30^. Our calculation results further show that the resultant dispersion curves with *w* = 13, and 33 mm for O-HMM [Figs. 2(b,c)] are very closed to those shown in Fig. 1(b), if a S-HMM is added with two different widths. We have calculated the dispersion curves with more different widths of S-HMM (not shown here), and the results suggest that the effect of the width of S-HMM on the dispersion curve can be neglected. Therefore, a tapered O-HMM waveguide with the top and bottom width as 13 and 33 mm is highly expected to keep high absorptance in the frequency bands (1)–(6) in Fig. 1(c). The remaining issue to be addressed is how to design a tapered S-HMM waveguide that absorbs efficiently in the frequency bands (7)–(11) in Fig. 1(c). On the one hand, the top and bottom widths of a tapered S-HMM waveguide should be properly chosen to make sure slow-light of TM_0_ mode cover the frequency range denoted by Δ*f* in Fig. 1(b), which is helpful to eliminate the low absorption band (7) in Fig. 1(c). On the other hand, the bottom width of tapered S-HMM waveguide has to be carefully selected to ensure the supported slow-light of higher TM order modes do not occur around the frequency bands (8)–(11) in Fig. 1(c). The optimization of the geometrical parameters of S-HMM waveguide is achieved by repeatedly calculating the dispersion curves of multiple waveguide modes for different widths. The dispersion curves of the optimized tapered S-HMM waveguide with 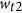 = 9 mm, and 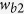 = 22 mm in Fig. 2(d,e) indicate the above-mentioned two conditions can be satisfied simultaneously.

**Figure 3:**
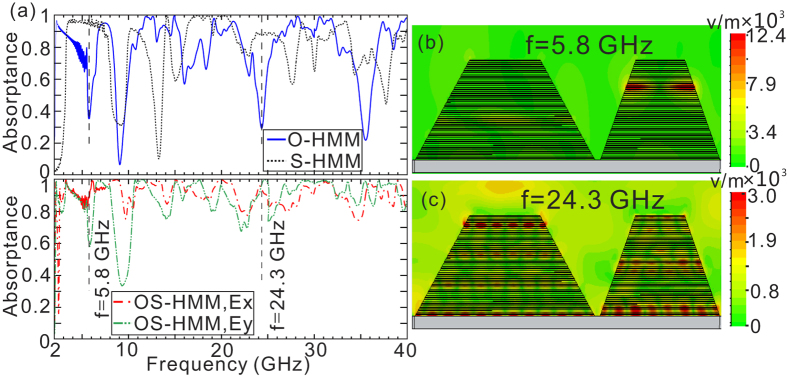
(**a**) The simulated absorption curves for O-HMM (blue line) and S-HMM (black line) waveguide arrays. The absorption curves of OS-HMM for different polarizations are represented by red (x polarization) and green (y polarization) lines, respectively. (**b**,**c**) The simulated amplitude distributions of electric field 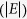 at 5.8 and 24.3 GHz, respectively, indicated by the vertical dashed lines in (**a**). The simulated geometrical parameters of the OS-HMM waveguide array in Fig. 2 are given as 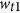 = 13, 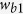 = 33, 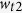 = 9, 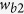 = 22, 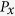 = 57, 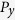 = 34, and *H* = 11.52 mm.

**Figure 4:**
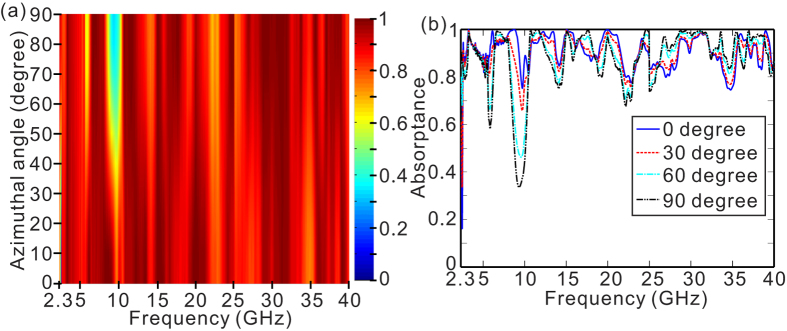
(**a**) The simulated absorptance of the OS-HMM waveguide array as a function of light frequency, and the polarization azimuthal angle with normal incidence. (**b**) The simulated absorption curves of the OS-HMM waveguide array for polarization azimuthal angle of 0, 30, 60, and 90 degree with normal incidence. All the geometrical parameters are the same as those in Fig. 3.

The simulated absorption curves of OS-HMM waveguide array for normal incident light further confirm the above-mentioned prediction [Fig. 3(a)]. In addition to keeping high absorptance in the frequency bands (1)–(6), it also presents the capability of overcoming the low absorptance around the frequency bands (7)–(11) by the tapered O-HMM waveguide array, resulting in a largely expanded absorption bandwidth ranging from 2.3–40 GHz. A considerable portion of EM wave is coupled into the TM_0_ mode in the S-HMM waveguide and finally localized at a specific position [see Fig. 3(b)], leading to a high absorptance around the frequency band (7). In the frequency bands (8)–(11), the EM wave is partially transferred to higher order slow-light modes of the S-HMM waveguide, but none of them locates at the bottom of the S-HMM waveguide [see Fig. 3(c) as an example], and hence effectively increase the attenuation and reduce the reflection by the metal substrate. It should be noted that the absorber is almost insensitive to the polarization of the incident EM wave in spite of the structural asymmetry. In order to verify the polarization insensitiveness, here we present the simulated absorptance of the OS-HMM waveguide array as a function of the polarization azimuthal angle with normal incidence [see Fig. 4(a)]. The polarization azimuthal angle is defined as the angle between the x axis and the incident polarization. As the polarization azimuthal angle increases from 0 to 90 degree, the absorption dips of the OS-HMM absorber becomes more apparent near 5.8, and 9.5 GHz. Except for the two absorption dips, the absorptance of the OS-HMM waveguide array is almost insensitive to the polarization within the whole frequency range of interest. To give a more clear comparison, we have presented the absorption curves for 4 different polarization azimuthal angles [see Fig. 4(b)]. The two absorption dips near 5.8 and 9.5 GHz becomes much sharper and broader with the increase of the polarization azimuthal angle. The absorptance with the frequency other than 5.8 and 9.5 GHz keeps high, indicating that the OS-HMM waveguide array presents less sensitiveness to the polarization azimuthal angle.

### Experimental Results and Discussion

**Figure 5:**
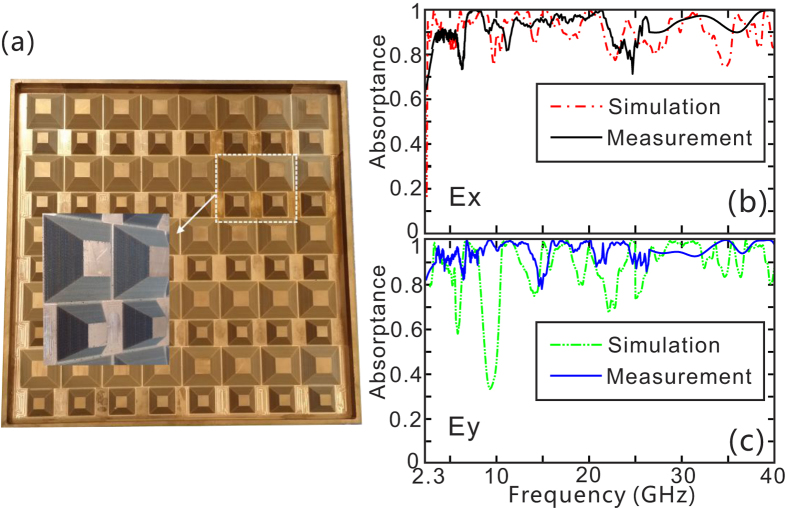
(**a**) The fabricated sample of OS-HMM absorber. (**b**,**c**) Simulated and measured absorption curves of OS-HMM absorber for (**b**) x polarization, and (**c**) y polarization. All the geometrical parameters are the same as those in Fig. 3.

**Figure 6:**
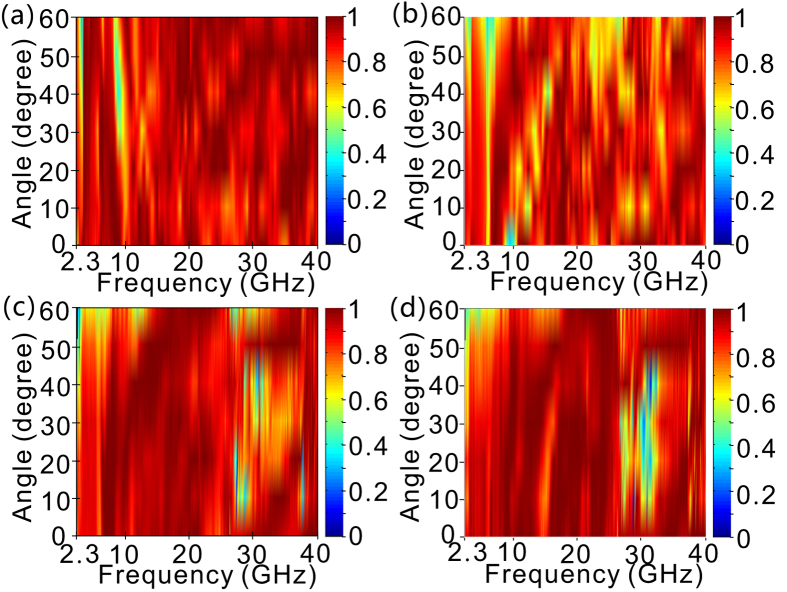
Dependence of simulated (**a**,**b**) and measured (**c**,**d**) absorptance of OS-HMM absorber on incident angle: (**a**,**c**) x polarization, and (**b**,**d**) y polarization.

Figure 5(a) shows the fabricated sample of a tapered OS-HMM waveguide array on a 300 mm × 300 mm copper substrate, which is used to suppress light transmission. As shown in Fig. 5(b,c), the OS-HMM absorber presents ultra-wide absorption bandwidth ranging from 2.3–40 GHz with high absorbance for both incident polarizations, which is basically consistent with the simulation result. And the slight discrepancy between the simulated and experimental absorption results might be caused by the fabrication error, and measurement precision due to the scattering. On the one hand, the structural precision is highly restricted by the radius of the milling cutter, which is 1 mm in our case. On the other hand, a small amount of scattering light from the OS-HMM absorber can not be received by the horn of the reflection test system, inducing that the measured absorptance is somewhat better than the simulation results. Figure 6 shows the influence of the incident angle on the absorptance of the OS-HMM absorber for two different polarizations. It can be seen that, despite the structural asymmetry of the designed absorber, it presents good polarization insensitivity even under incident angle of 60°. The absorptance almost keeps high (above 80%) even as the incident angle increases to 60° for most portion of radio frequency spectrum, implying excellent independence of the incident angle.

In conclusion, we have proposed an ultra-broadband microwave absorber comprised of two different-sized tapered HMM waveguide arrays, each of which works at wide but different multiple absorbing bands. In this strategy, properly selecting the geometrical parameters for each waveguide can connect different absorption bands of different waveguides, effectively widening the total absorption bandwidth. We demonstrate numerically and experimentally that the proposed absorber shows a strong absorption within the ultra-wide absorption bandwidth of 2.3–40 GHz with almost independence of polarization and incident angle. The results presented here hold a great promise for designing practical metamaterial absorbers with the applications targeted in stealth, camouflage, and antenna at microwave frequencies.

## Methods

### Numerical modeling

The simulated absorptance of all patterned multi-layered structures shown in Figs 1, 3, 4, 5 and 6 are conducted by CST MICROWAVE STUDIO 2012. The dispersion curves in Figs 1 and 2 are calculated as the Bloch mode propagates along z-direction with a commercial finite difference time domain simulation software (Rsoft). For periodical optical structures, the dispersion relations are calculated as only one unit cell is set in the simulation, and the periodical boundary conditions are set along x, y, and z-directions. The unit cell of the uniform HMM waveguide array is shown in Fig. 1(a). The lattice constants along x and y-direction are all set to be 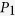 = 34 mm. *P* represents the lattice constant 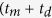, 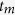 = 0.04 mm, 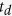 = 0.2 mm) along z-direction. The unit cell of the uniform OS-HMM waveguide array is presented in the right side of Fig. 2(a). The dispersion relations of the uniform O-HMM (S-HMM) waveguide shown in Fig. 2(b–e) are calculated for the O-HMM (S-HMM) waveguide models. The lattice constants along x-direction, y-direction and z-direction are assumed to be 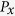 = 57 mm, 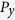 = 34 mm, and *P* = 0.24 mm, respectively. The electric conductivity of copper is assumed to be *σ* = 5.8 × 10^7^ s/m, and the permittivity of lossy dielectric spacer (FR4) is set to be *ε* = 4.3 + 0.025i.

### Sample fabrication and measurement

Each copper layer with a thickness of 0.04 mm is printed on a FR4 layer with a thickness of 0.15 mm by the method of multilayered printed circuit board. The composite layers are then sandwiched using adhesive with a thickness of 0.05 mm (almost the same dielectric constant as FR4). A standard mechanical milling method is followed to form the OS-HMM absorber. The absorptance measurement of the fabricated absorber is carried out using a NRL-arch reflection test system^33^ equipped with a vector network analyzer (Agilent Technologies, N5230).

## Additional Information

**How to cite this article**: Yin, X. *et al.* Ultra-wideband microwave absorber by connecting multiple absorption bands of two different-sized hyperbolic metamaterial waveguide arrays. *Sci. Rep.* **5**, 15367; doi: 10.1038/srep15367 (2015).
